# Effectiveness of a new 3D printed simulator for mitral transcatheter edge-to-edge repair in enhancing the confidence and procedural skills of the operator

**DOI:** 10.1186/s41205-024-00230-1

**Published:** 2024-08-05

**Authors:** Angel Babu, Michele Bertolini, Michael Mullen, Andrew Cook, Aigerim Mullen, Claudio Capelli

**Affiliations:** 1 Institute of Cardiovascular Science, University College London, London, WC1E 6BT UK; 2 Department of Mechanical Engineering, Politecnico di Milano, Milan, 20133 Italy; 3 Barts Health NHS Trust, London, E1 1BB UK; 4 Abbott Structural Heart UK and Ireland, Blyth Valley Business Park, Solihull, B90 8AJ UK

**Keywords:** Mitral valve (MV), Mitral regurgitation (MR), Mitral transcatheter edge-to-edge repair (m-TEER), MitraClip™, 3D printing, Surgical training, Anatomical simulators

## Abstract

**Background:**

. Mitral transcatheter edge-to-edge repair (m-TEER) is a minimally invasive procedure for treating mitral regurgitation (MR). m-TEER is a highly technical procedure, and a steep learning curve needs to be overcome for operators to ensure optimal patient outcomes and minimise procedural complications. Training via online simulation and observation of procedures is not sufficient to establish operator confidence; thus, advanced hands-on training modalities need to be explored and developed.

**Methods:**

. In this study, a novel anatomical simulator for m-TEER training was evaluated in comparison to a standard model. The proposed simulator resembled the anatomical features of the right and left atrium, left ventricle and mitral valve apparatus. Participants in the questionnaire (*n* = 18) were recruited across 4 centres in London with (*n* = 8) and without (*n* = 10) prior experience in m-TEER. Participants were asked to simulate procedures on both an idealised, routinely used simulator and the newly proposed anatomical model. The questionnaire was designed to assess (i) participants’ confidence before and after training and (ii) the realism of the model in the context of the m-TEER procedure. The results of the questionnaires were collected, and statistical analysis (t-test) was performed.

**Results:**

. Both models were equally beneficial in increasing operator confidence before and after the simulation of the intervention (*P = 0.43*). However, increased confidence after training with the anatomical model was recorded (*P = 0.02*). Participants with prior experience with m-TEER therapy were significantly more confident about the procedure after training with the anatomical model than participants who had no prior experience (*P = 0.002*). On average, all participants thought that the anatomical model was effective as a training simulator (*P = 0.013*) and should be integrated into routine training (*P = 0.015)*). Participants with experience thought that the anatomical model was more effective at reproducing the m-TEER procedure than the idealised model (*P = 0.03*).

**Conclusions:**

. This study showed how a more realistic simulator can be used to improve the effectiveness of m-TEER procedural training. Such pilot results suggest planning future and large investigations to evaluate improvements in clinical practice.

**Supplementary Information:**

The online version contains supplementary material available at 10.1186/s41205-024-00230-1.

## Background

Mitral regurgitation (MR) is characterised by the systolic retrograde flow from the left ventricle (LV) to the left atrium (LA) [1]. MR is a growing public health problem in Europe being the second most prevalent valvular heart disease necessitating a surgical approach to prevent heart failure [1]; [2]. The occurrence of MR is thought to increase but remains widely underdiagnosed [3]; [4].

As patients with functional MR are largely composed of an aging population with multiple comorbidities, medical therapy is focused on treating primary disease (such as heart failure etc.) or alleviating symptoms. Surgical treatment, such as mitral valve (MV) repair, is the intervention of choice and is overall associated with lower operative mortality than MV replacement [5]; [6]. However, many patients remain ineligible for such treatment due to the high surgical risk [2]. Minimally invasive procedures, such as mitral transcatheter edge-to-edge repair (m-TEER), have been proven to be effective alternatives in selected patients [7]. M-TEER was conceived to mimic the efficacy of the surgical repair proposed by Ottavio Alfieri, where the anterior and posterior leaflets of the MV are sutured by a surgical stitch creating a double orifice valve [8], therefore reducing MR [9]. To date, more than 200,000 patients have undergone minimally invasive procedures with the MitraClip™ system (Abbott Laboratories, Santa Clara, CA, USA), the most widely employed m-TEER device [10]. Pre-procedural planning is conducted using 3D TEE and transthoracic echo, which assess MV morphology and review clip implantation strategy to avoid complications [11]. Procedure-related complications can include atrial or ventricular perforation, pericardial effusion, cardiac tamponade and the persistence of an iatrogenic atrial septal defect. Device-related complications can include persistent MR, leaflet injury and chordae rupture [12].

M-TEER results are directly influenced by operators’ skills and experience [13]. Residual MR is associated with increased risk of mortality after MitraClip™ implantation. Hence, the decision-making during the procedural steps of the intervention assumes a crucial importance to tackle the challenges of the transeptal puncture, the insertion of a steerable guide catheter, and the positioning of the clip. M-TEER is still associated to a steep operator learning curve. A multicentre study that analysed over 12,000 procedures performed at over 275 sites concluded that significant improvements in optimal procedural outcomes, decreased procedural timing and complications were notable on learning curves after approximately 50 cases, and continued improvements were visible after up to 200 cases [14]. Navigating the delivery system within the LA while preventing contact with the left atrial wall and valve tissue, coupled with grasping leaflets and evaluating sufficient reduction in MR, has been reported to be the most challenging aspect of m-TEER [15]. Importantly, the standardization of m-TEER procedure is possible only when dealing with more simple anatomies and in case of beginner operators. Increasing the confidence with this tool through a specific training program can widen the spectrum of pathologies that can be treated [13].

Learning modalities in addition to “on-the-job” training are necessary due to the limited working hours available to attain adequate proficiency in m-TEER procedures [16]; [17]; [18]. In the context of general cardiac training, 3D-printed anatomical replicas have been increasingly used as an ex vivo training tool to enhance preoperative planning and decision-making [19]. 3D-printed models could circumvent the reliance on fluoroscopic guidance, mimic the dimensions and boundaries of the cardiac structures and facilitate storage and transport [20]. Despite the potential advantages of 3D printing adoption, to date, anatomy-based simulators specifically designed for m-TEER training are scarce and are mainly proposed to simulate specific steps of the procedure [21] and their effectiveness still requires further evidence [22].

The aim of this study was to explore the benefits of a 3D printed, anatomically realistic simulator for the m-TEER procedure to facilitate the acquisition of procedural skills and increase operator confidence among involved participants.

## Methods

The study was designed to assess the effectiveness of a 3D-printed, anatomically realistic simulator in comparison to a standard, idealised model for device implantation in training for the m-TEER procedure. The learning experience was assessed via a questionnaire before and after the training procedure.

### Participants

Participants were recruited across 4 different centres in London (UK), namely, St. Bartholomew’s Hospital, Royal Brompton Hospital, Zayed Centre for Research, and Cleveland Clinic London Hospital. Prior to the experiment, all the participants were given instructions about the experiment and the equipment to be used. Guidance on all controls and functions of the system was provided, as well as a practical demonstration prior to the timed m-TEER simulation procedure.

### Equipment

**Fig. 1:**
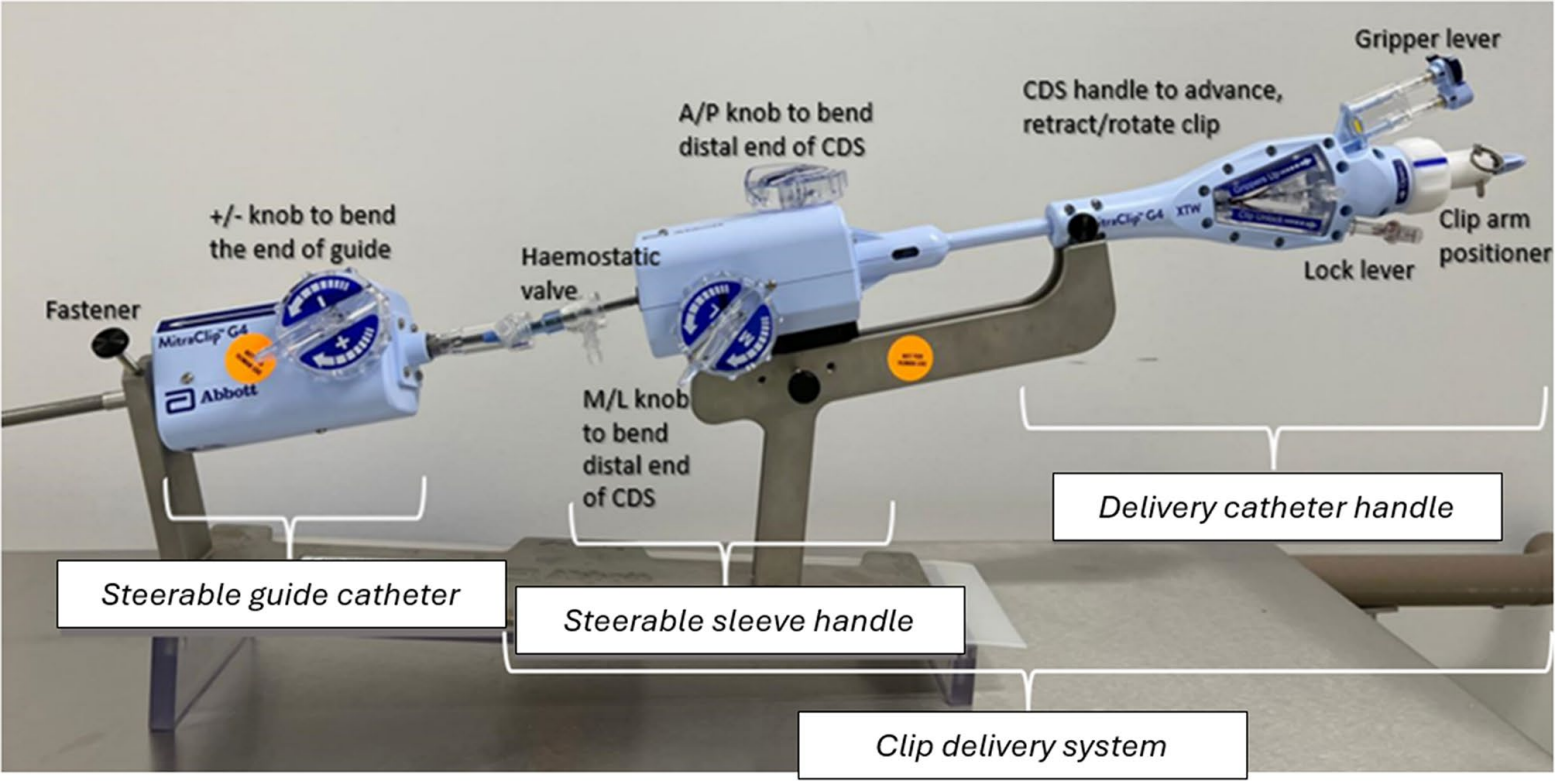
Components of the MitraClip™ system, including the clip delivery system (CDS) and the steerable guide catheter (SGC)

The MitraClip™ G4 system, used in this experiment to simulate the m-TEER procedure, consists of two parts: (i) the steerable guide catheter (SGC), used to introduce the whole procedural device to the patient and navigate through the transseptal puncture; and (ii) the clip delivery system (CDS), which has an implant preloaded and used to deliver, position, and deploy the clip (Fig. 1). The CDS is composed of a delivery catheter, a steerable sleeve, and a mechanical clip (implant), and its components are made of cobalt-chromium alloy (clip arms) and nitinol (grippers with the frictional elements) sheathed in polyester fabric. Clip arms and grippers are manipulated using controls on the CDS.

The m-TEER procedure was simulated and assessed with two subsequent training systems: (i) an idealised implantation model; and (ii) an anatomically realistic implantation model.

**Fig. 2:**
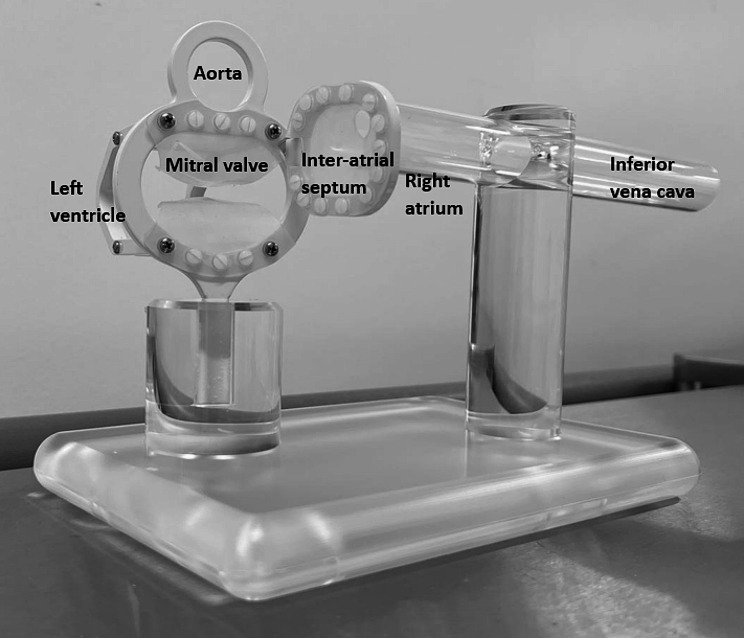
Photograph of the idealised implantation model. This device was made of a rigid polymeric tube to allow the insertion of a guide catheter mimicking the route from the inferior vena cava to the interatrial septum (IAS). At the end of the tube, a membrane with three holes mimicked the access through the IAS. A rigid elliptical plastic ring holds two silicone membranes to simulate the mitral valve leaflets. Downstream, rigid U-shaped arms are connected to the MV ring to define the left ventricle contour

The idealised implantation model (Fig. 2), routinely used for MitraClip™ training (Abbott Laboratories, Santa Clara, CA, USA), is made of a rigid, tubular polymeric part to allow the insertion of a guide catheter mimicking the route from the inferior vena cava (IVC) to the inter-atrial septum (IAS). At the end of the tube, a membrane with three holes mimicked the access through the IAS. Placed orthogonal to the IAS, a rigid elliptical plastic ring holds two silicone membranes to simulate the valve leaflets. Downstream, rigid U-shaped arms are connected to the MV ring to define the LV contour.

**Fig. 3:**
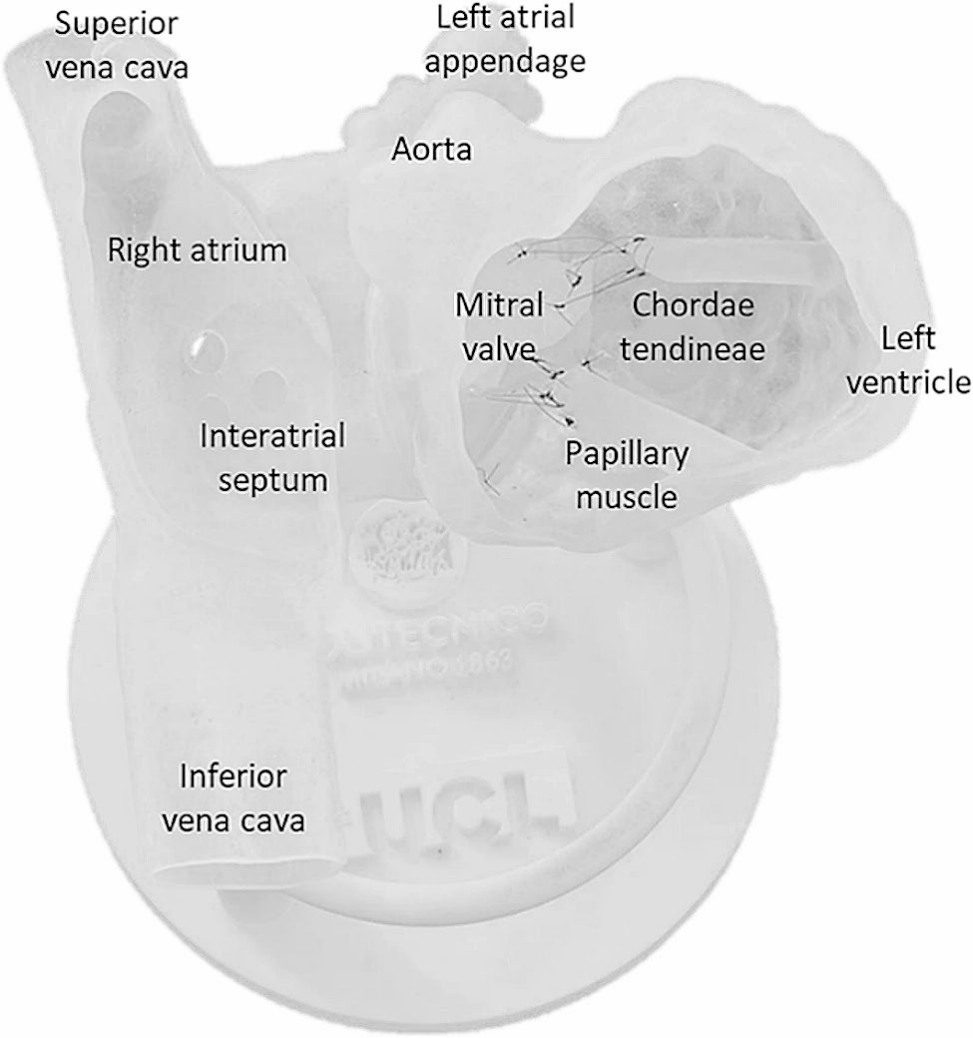
Photograph of the anatomically realistic model with details of the features included

The anatomically realistic model (Fig. 3) was previously designed and manufactured by our group [23]. The digital model of a heart (atria and LV) was generated by segmenting computed tomography (CT) starting from the blood pool and subsequently modified for *i*) adding anatomical features not fully visible with CT, *ii*) adapting the model to interact with the m-TEER equipment, and *iii*) ensuring modularity of the system. Modifications included modelling by extrusion portions of PMs, creating 22 Fr transseptal holes to simulate common transeptal puncture locations, opening windows in the model to allow a clear view of the implantation site and designing a solution to make the MV replaceable [23].

Additionally, a base consisting of 3 columns interconnected by a toroid was designed to align and stabilise the cardiac structure in a way that replicates the position and angulation of the heart during the procedure. The model was manufactured with a Polyjet technology 3D printer (Stratasys, Eden Prairie, USA) using commercial VeroClear (rigid) and Agilus30 Clear (soft) resins. Chordae tendineae were also replicated in the model using polypropylene sutures to connect the distal tips of the MV to the heads of PMs [23].

### Procedure

**Fig. 4:**
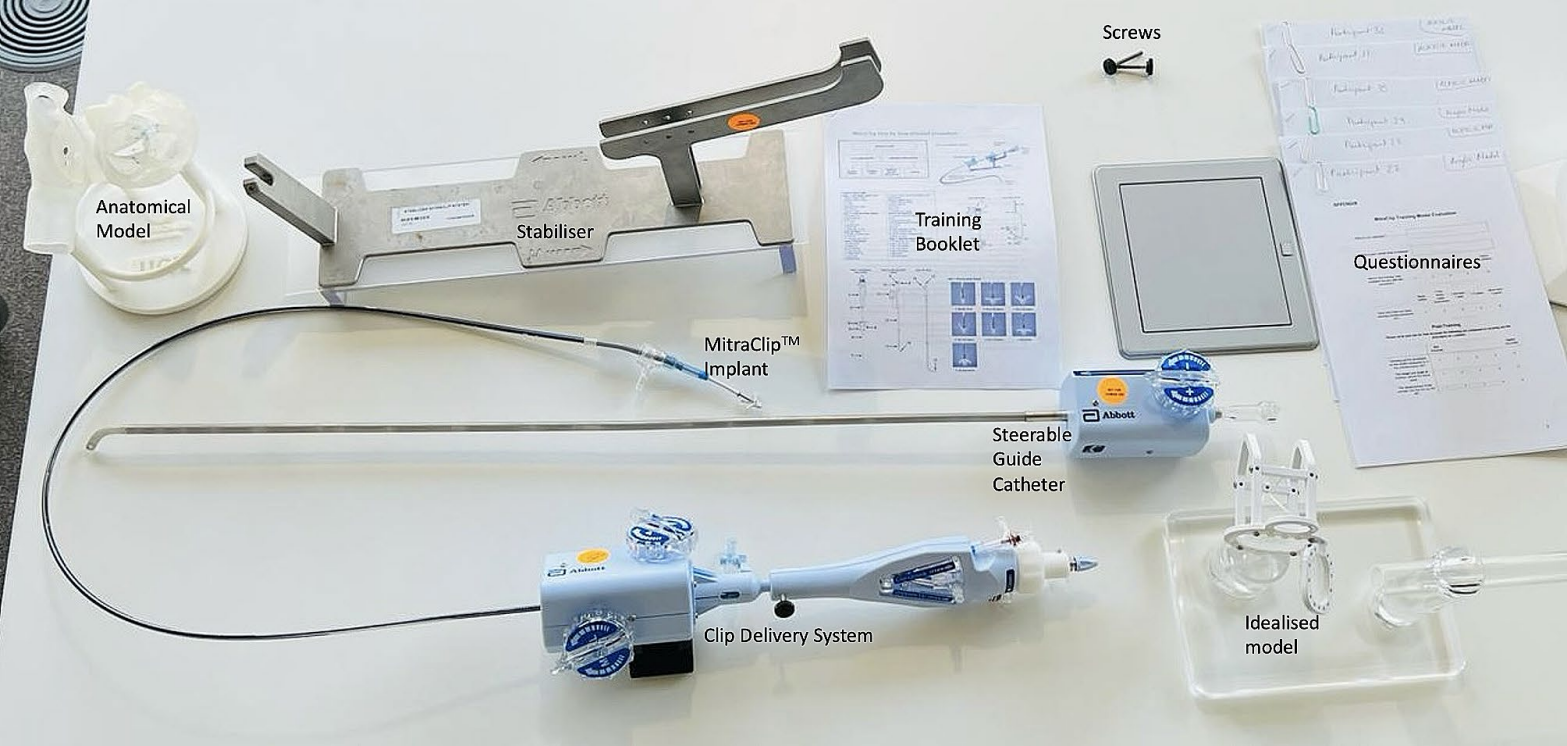
Labelled layout of the MitraClip^™^ system and training equipment

The MitraClip™ demo kit and both models were placed on a table (Fig. 4). Participants completed the tasks through direct visualisation of the models and had a trained MitraClip™ proctor verbally translating movements made by the guide catheter and MitraClip™ implant within the models. Each participant was asked to practice the insertion of the clip device first in the idealised simulator and then in the anatomical simulator by completing the following tasks:

1. Insertion of the SGC through the IVC and into the RA;
2. Transseptal crossing into the LA and insertion of the CDS;
3. Orientation in the LA and advancing CDS through the mitral valve annulus (MVA);
4. Aligning the clip perpendicular to the coaptation of the MV leaflets in the A2P2 segment of the valve;
5. Grasping leaflets using grippers and assessment of adequate leaflet capture;
6. Clip closure.

### Data collection

A questionnaire was designed to record the knowledge and confidence of the respondents before and after each implantation test. Questions were asked to assess: (1) operator confidence in the skills necessary for the procedure; (2) the accuracy and realism of each model practice in comparison to the real m-TEER procedure and (3) the perceived benefits of introducing the model into training. The questionnaire consisted of 18 questions answered on a 5-point Likert scale and 2 open questions, and the questionnaire is available in the Supplementary Materials. Additionally, operator confidence was assessed for each stage of the procedure using a 5-point Likert scale and the sum was calculated. Qualitative feedback on positive aspects and future improvements of the training models was reported by the participants in the form of statements. Responses were collected anonymously, and identifiable data were not collected. The performance of each participant was timed from insertion to clip deployment.

### Statistical analyses

T-tests (two-tailed and paired) and confidence interval (CI) evaluations were performed on the collected data to assess the performance and the differences between participants. All analyses were completed using Excel spreadsheet software (Microsoft, Redmond, USA).

## Results

### Demographic data

A total of 18 participants completed the proposed m-TEER simulation procedures and completed the questionnaire. Among them, 10 clinicians did not have any prior clinical experience with m-TEER procedures and 8 were not proceduralists. The positions held by the participants at the time of the experiment are recorded in Table 1.

**Table 1:** Summary of participant experience with performing TEER simulations and their grade or professional title

Years of experience	Number of participants (*n*)	Doctor grade/ title
*No prior experience*	10	Radiologists (*n* = 3) Interventional cardiologists (*n* = 2) Cardiac surgeon (*n* = 1) Cardiac physiologist (*n* = 1) Cardiac consultant (*n* = 1) Cardiac registrar (*n* = 2)
*Less than 1 year*	2	Interventional cardiologists (*n* = 2)
*1–5 years*	2	Interventional cardiologists (*n* = 1) Cardiac consultant (*n* = 1)
*More than 5 years*	4	Cardiac consultants (*n* = 2) MitraClip™ proctors (Abbott certified procedural therapy specialist) (*n* = 2)

### Outline of the simulation procedure

**Fig. 5:**
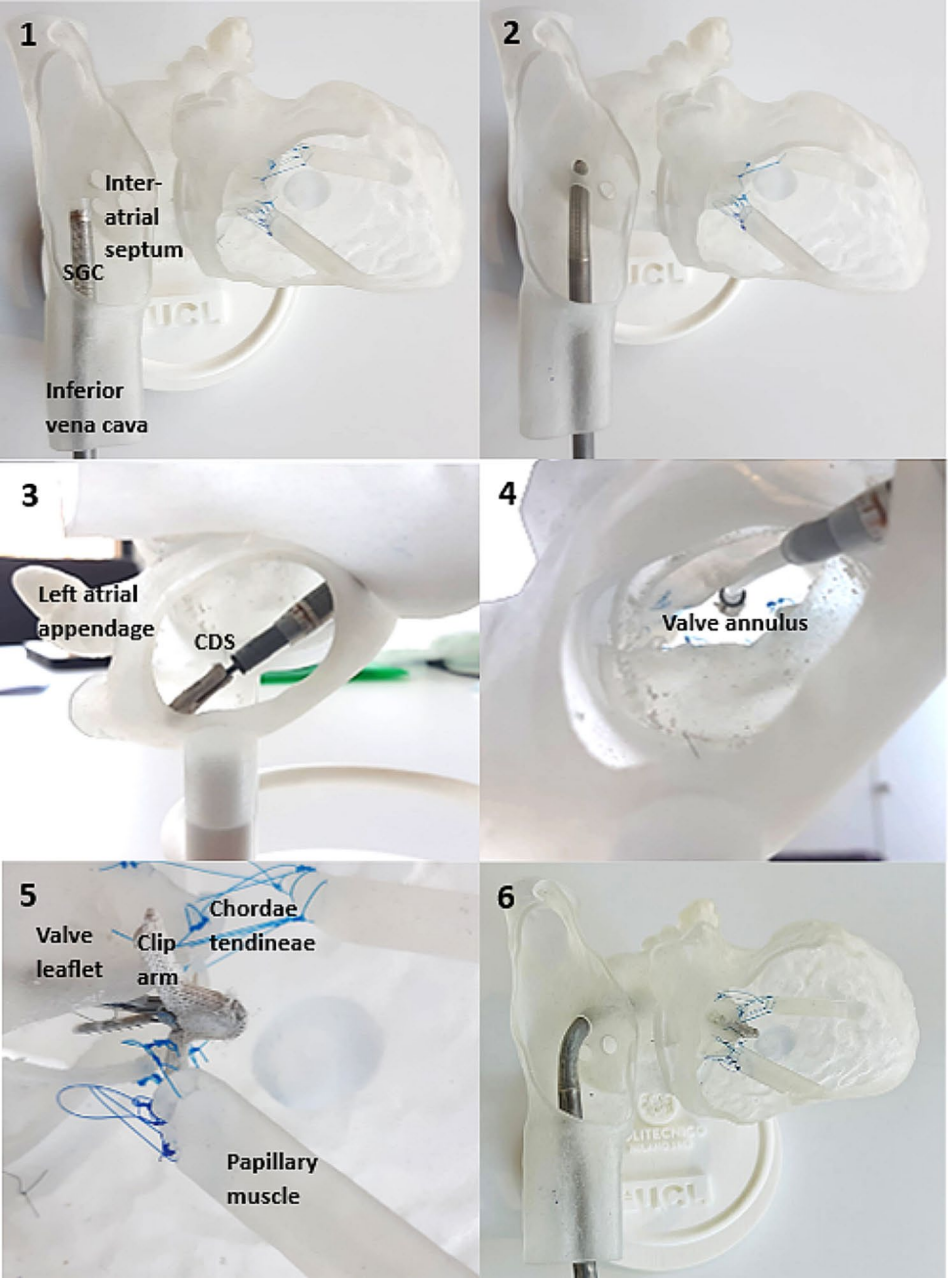
The six steps of the simulated procedure on the 3D printed model: (**1**) Insertion of the SGC into the RA and the IVC; (**2**) Transseptal crossing into the LA and insertion of the CDS; (**3**) Orientation in LA; (**4**) Advancing the CDS through the MVA and ensuring the alignment of the clip perpendicular to the coaptation of the MV leaflets; (**5**) Grasping leaflets using grippers and assessment of adequate leaflet capture; (**6**) Closing the clip

**Fig. 6:**
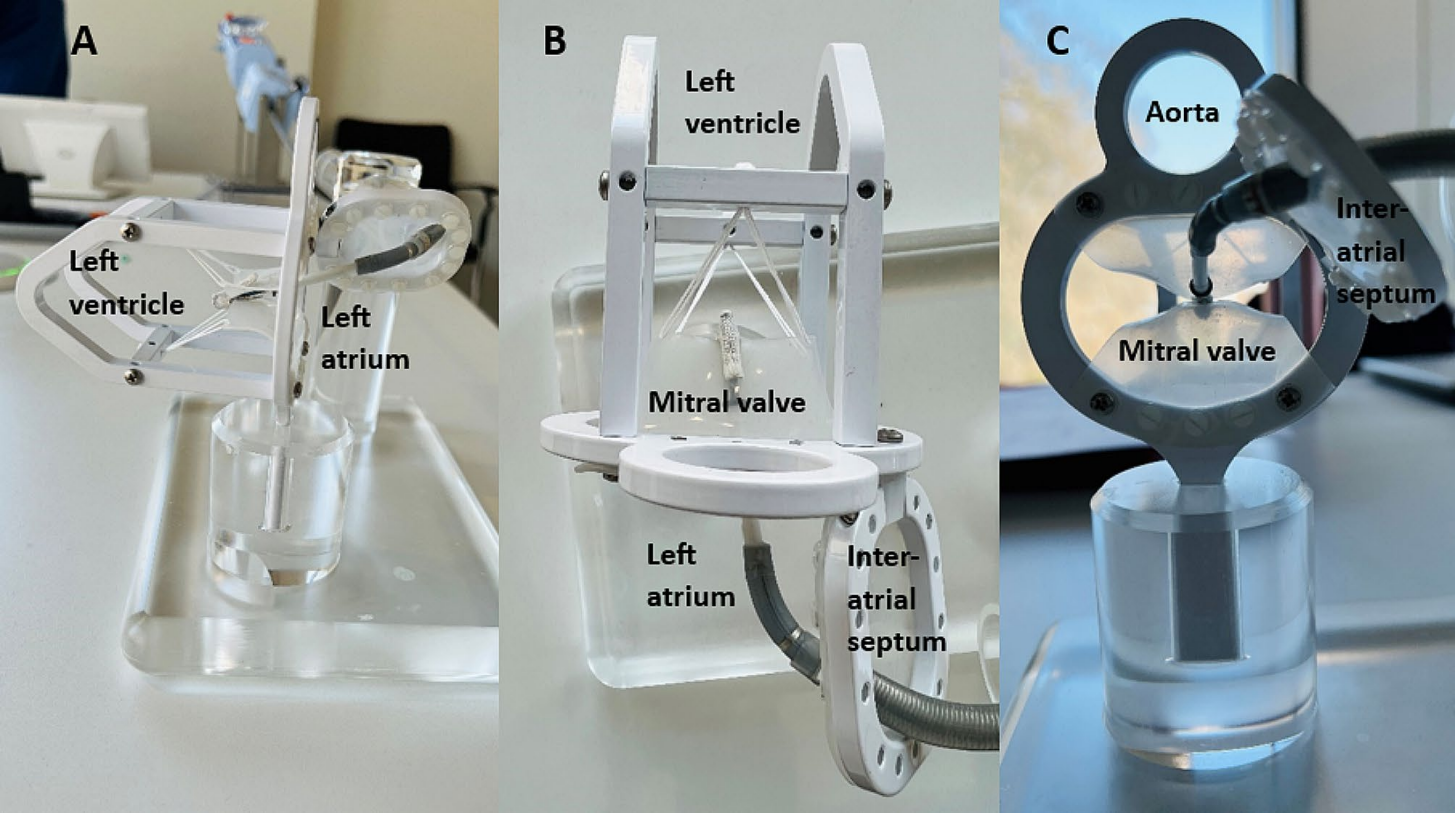
Completion of the training task on the idealised model through different perspectives: (**A**) Lateral view; (**B**) Top view; and (**C**) Caudal view

Figure 5 depicts the six stages of the simulated procedure in the anatomical model. The training procedure was completed successfully when all the participants implanted the device. Figure 6 shows the MitraClip™ device implanted in the idealised model.

### Timing of the simulation procedures

**Fig. 7:**
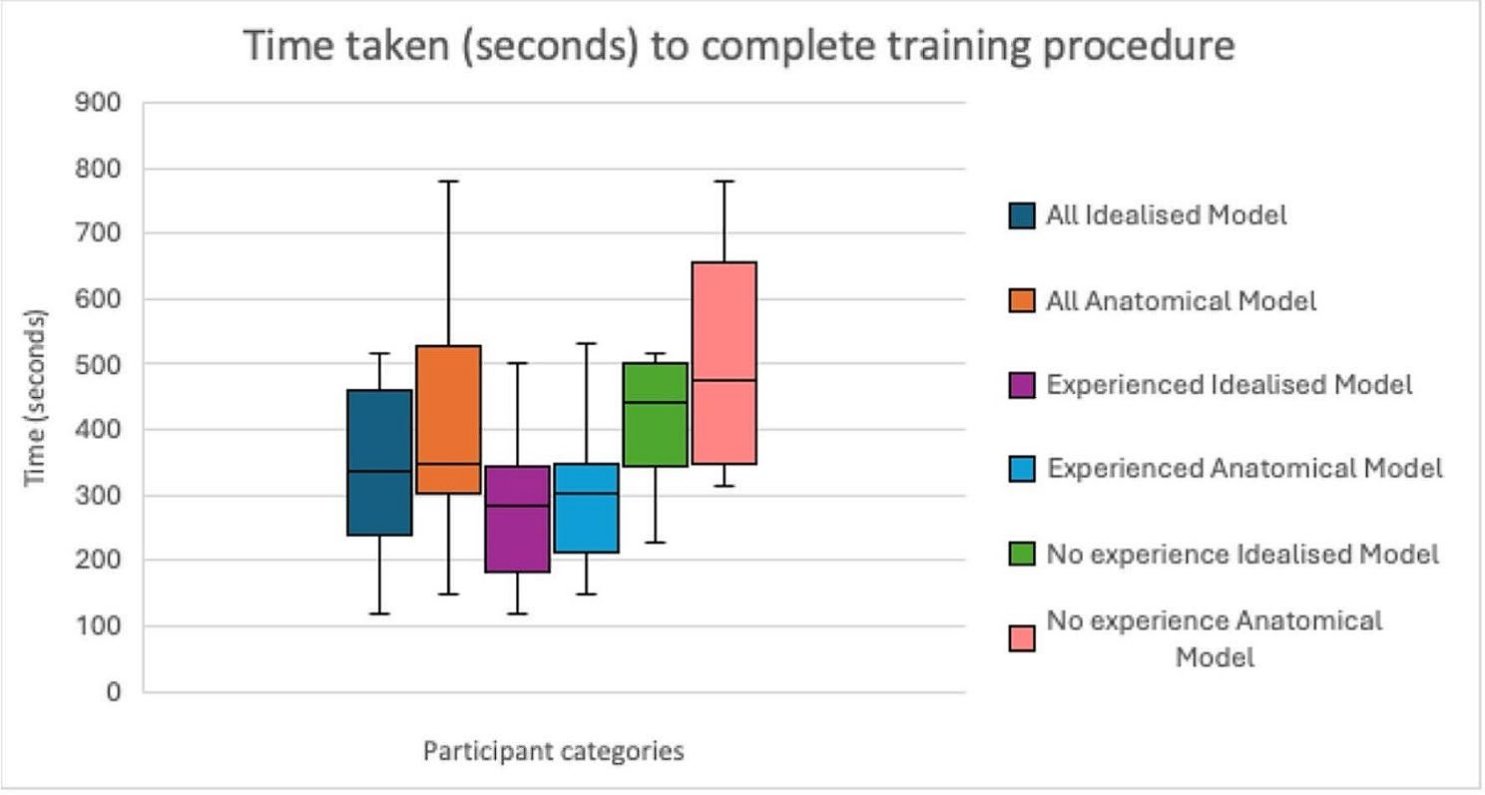
Duration of training with the idealised and anatomical models. The first two box plots from the left depict the distribution of timings by all participants. The middle two box plots show the distribution of timings of participants with prior experience in m-TEER. The last two box pots on the right show the distribution of time taken to complete the training task by participants with no prior experience with m-TEER

The average time for completing the m-TEER training tasks was 372 (± 176) seconds for the idealised model and 403 (± 174) seconds for the anatomical model (Fig. 7). Overall, no significant difference in timing between the two training models was observed among the participants overall (*P =* 0.34). Clinicians with prior experience in performing m-TEER were significantly faster at using both the idealised (*P = 0.018*) and anatomical (*P = 0.008*) models than clinicians without experience.

### Effects on participant confidence

Participants rated their confidence in doing this procedure in vivo before training with the idealised and after training with both models. Overall, an increase in confidence was achieved from 2.70 (± 1.61) before training to 3.20 (± 1.11; *P =* 0.14) and 3.40 (± 1.06 *P =* 0.02) after training with the idealised and anatomical model, respectively (Table 2). Before training, the average scoring of confidence was significantly higher among clinicians with experience 4.25 (± 0.70) than participants with no prior experience, scoring 1.30 (± 0.70) (*P < 0.001*). Participants with prior experience scored significantly higher after training with the anatomical model, 4.13 (± 0.60), than participants with no prior experience, 2.77(± 1.00) (*P =* 0.002).

**Table 2:** Average scores of participant confidence in performing the m-TEER procedure. The items were scored on a 5-point likert scale (1 – not confident & 5 – highly confident)

Participant category	Before training	After idealised model	After anatomical model
*All participants*	2.70 ± 1.61	3.20 ± 1.11	3.40 ± 1.06
*Participants with experience*	4.25 ± 0.70	3.75 ± 1.00	4.13 ± 0.60
*Participants with no prior experience*	1.30 ± 0.70	2.88 ± 1.10	2.77 ± 1.00

**Fig. 8:**
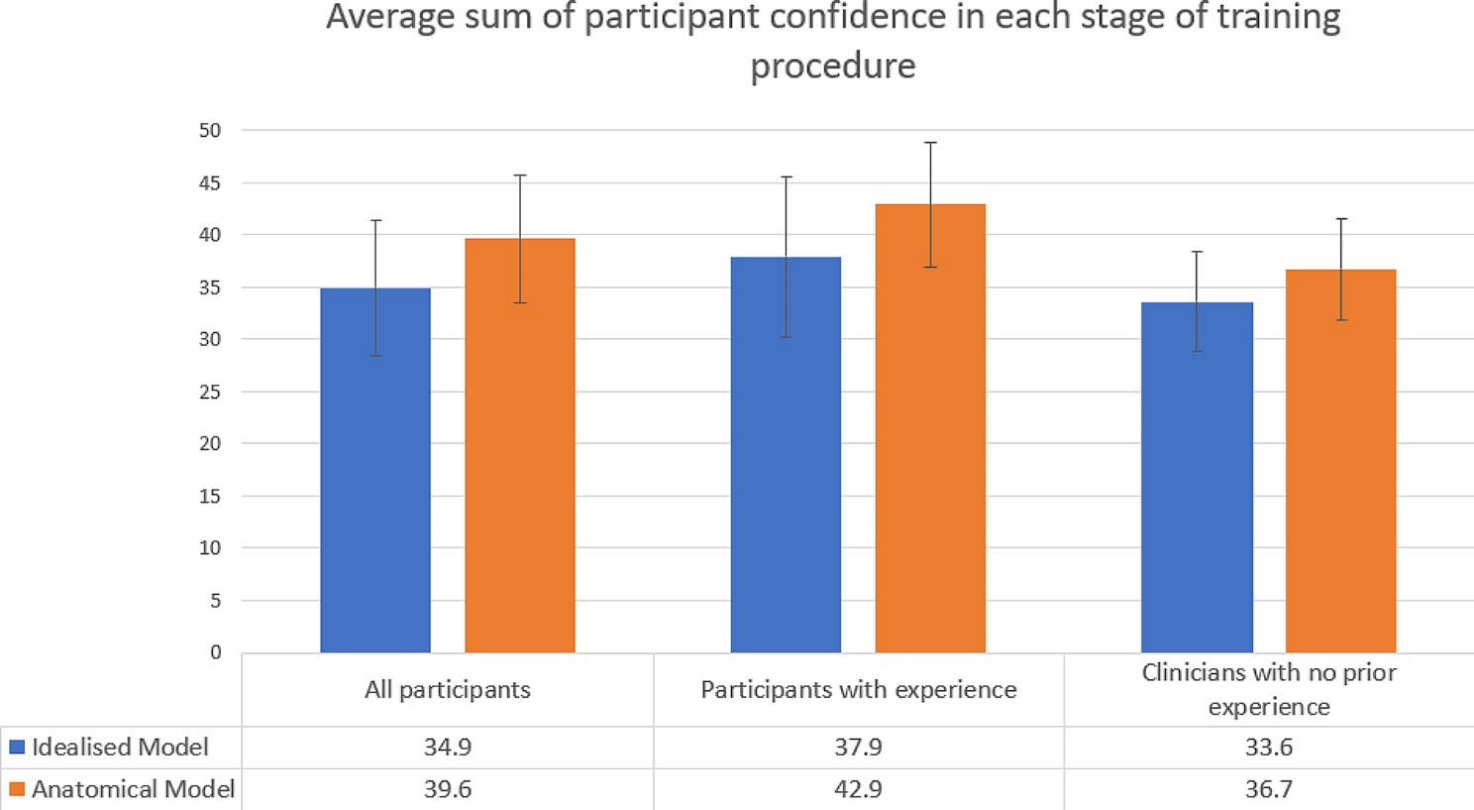
The sum of the confidence values for each stage of the m-TEER procedure after training with each model for all participants and participants with and without prior experience in m-TEER (maximum score of 50)

Overall, participants rated a greater sum of confidence across the stages of the m-TEER training procedure for the anatomical model than for the idealised model (*P = 0.003*) (Fig. 8). Participants with prior experience rated a greater sum of confidence after training with the anatomical model than participants without prior experience in m-TEER (*P* = 0.016).

The training on the anatomical models after having trained on the idealised model, helped the participants gain significantly greater confidence in recognising areas of interest on the MV, assessing the position and orientation of the clip above the valve, and reopening and repositioning the clip (Table 3). The skill with the lowest confidence rating among all participants was transseptal crossing, for both training models. Participants with no prior experience in m-TEER rated lower scores of confidence for steps requiring manipulation of the CDS.

**Table 3:** Average score of confidence for each step of training with the idealised model and the anatomical model. P values compare scores between idealised and anatomical models

Steps of the m-TEER procedure	Idealised model	Anatomical model	T-test *P* values
Transseptal crossing	3.3 (± 0.91)	3.5 (± 0.72)	0.43
Steering clip in LA	3.6 (± 0.78)	3.9 (± 0.78)	0.10
Positioning trajectory of the clip	3.6 (± 0.98)	3.9 (± 0.78)	0.33
Recognising area of interest on MV	3.4 (± 1.2)	4.0 (± 0.87)	< 0.01
Assessing position of clip above valve	3.6 (± 0.78)	4.1 (± 0.75)	< 0.01
Assessing orientation of clip above valve	3.6 (± 0.86)	4.1 (± 0.80)	0.01
Grasping leaflets	3.7 (± 0.71)	4.2 (± 0.73)	0.46
closing a clip	4.0 (± 0.69)	4.2 (± 0.90)	0.46
Re-opening clip and re-positioning	3.6 (± 0.78)	4.1 (± 0.86)	< 0.01

### Participants’ ratings

All participants rated the anatomical model as more effective at reproducing the key steps and challenges of the m-TEER procedure, with an average score of 3.5 ± 0.94 (out of 5) for the idealised model in comparison to the anatomical model 4.2 ± 0.73 (*P =* 0.013). Also, participants rated significantly higher for the anatomical model to be integrated into training (*P =* 0.014), with a score of 3.9 ± 0.78 (out of 5) with the idealised model, in comparison to 4.4 ± 0.51 with the anatomical model.

**Fig. 9:**
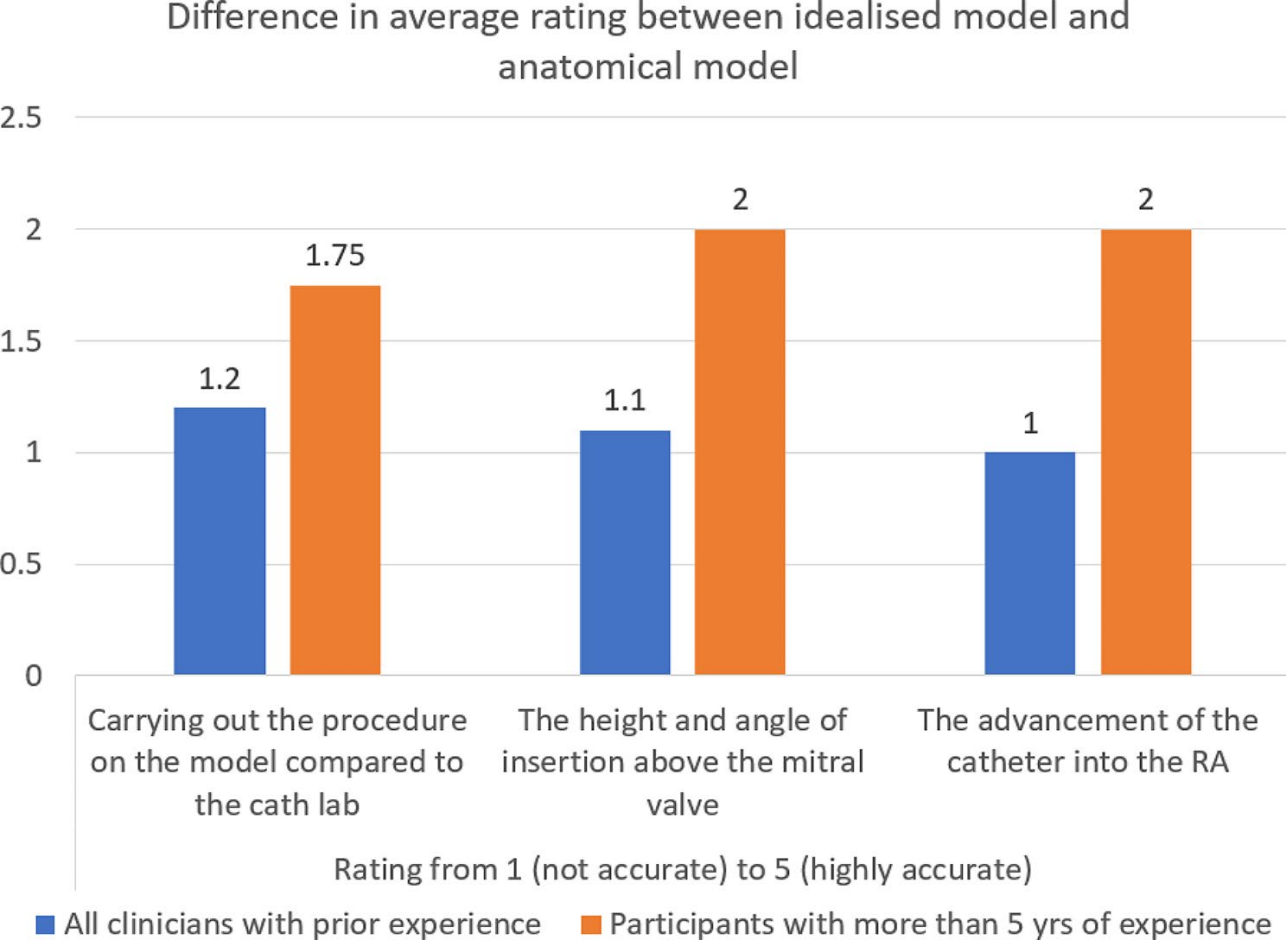
Differences in the scores of all participants with experience and highly experienced participants with more than 5 years of training in m-TEER scoring statements: (1) “How accurate is carrying out the procedure on the model compared to the catheterisation lab?”, (2) “How accurate is the height and the angle of insertion above the mitral valve?” and (3) “How accurate is the advancement of the catheter into the right atrium”. Positive results indicate that the anatomical model had a higher rating than the idealised model

Then, solely participants with prior experience with m-TEER rated three statements regarding accuracy and realism of both models in comparison to performing m-TEER in vivo (Fig. 9). Participants with prior experience rated significantly greater accuracy in carrying out the training on the anatomical model than the idealised model for performing the m-TEER procedure in the catheterisation lab (*P* = 0.019). Additionally, participants with the most experience rated the anatomical model as more accurate and realistic to the m-TEER procedure in vivo, which scored on average 4 ± 0.53 (out of 5) in comparison to 2.8 ± 1.16 with the idealised model. Participants with the most experience rated significantly greater accuracy for the advancement of the catheter into the RA in the anatomical model (*P* = 0.016) in which the idealised model scored 1.5 ± 0.58, in comparison to the anatomical model which scored 3.5 ± 1.29.

### Qualitative feedback

Feedback about the idealised model reiterated its usefulness in “orienting” and “familiarising” oneself with the MitraClip™ system, as it was “easy to use”. The anatomical model was reported to be “realistic”, “accurate” and “representative” of the “human anatomy”. Among the suggested improvements (Table 4), a common suggestion for both models was the need to implement visual feedback.

**Table 4:** Table highlights future key improvements suggested by participants for the idealised and anatomical models. Quotation marks denote text directly taken from participants’ feedback, while unquoted text is a paraphrased synthesis of participants’ responses

Idealised model	Anatomical model
“Leaflets and IVC” need to be sturdier and “concrete”	“Transseptal puncture holes are not easy for manoeuvring with steerable guide catheter”
“Also, ability to choose angles to simulate challenging anatomy”	Attachment of camera visualisation to system for improved visualisation. “Cameras to replicate 3D echo views of bicomissural, LVOT and LA views”
“Not representative of anatomy” seen in fluoroscopy	“Patient-specific pathology in valves”
“Attachment of camera visualisation to system for improved visualisation. Cameras to replicate 3D echo views of bicomissural, LVOT and LA views”	“More suited to those with more confidence or experience in controlling the MitraClip™”
“3D modelling could develop more realistic parts”, to replicate intra- and extracardiac structures	“Current mitral valve leaflets are paediatric and should be representative of normal adult mitral valves with ant. Leaflet being 1/3 in circumference and post. Leaflet 2/3 in circumference”
More grip at the bottom to prevent slipping	“Mitral valve mobility could be improved”

## Discussion & conclusions

This research aimed to investigate the effectiveness of training models for the m-TEER procedure. Specifically, we sought to determine whether the use of an anatomical model could enhance the training experience compared to an idealised setup. The findings demonstrated that the anatomical model could contribute to enrich the experience of proceduralist and support a more effective acquisition of the necessary skills for each step of m-TEER procedural training, leading to increased operator confidence. Participants expressed a stronger preference for the anatomical model. However, variations were observed among the participant subgroups. Those with prior m-TEER experience exhibited meticulous adherence to experimental guidelines, while those without prior experience showed a tendency to advance the catheter guide with less consideration for intracardiac structures. This emphasises the need for training to be stratified by the level of experience. Thus, tailoring instructional strategies to build on the background knowledge of participants can enable them to progress up the learning curve from mentally assembling a step-by-step sequence to execute the various stages of the procedure.

The idealised model was found to be more effective in training due to its simplicity, particularly in building confidence in manipulating the knobs on the system and comprehending how the movements translate to intracardiac structures. A large proportion of written feedback was closely linked to “familiarising” themselves with the device, particularly in participants with no prior experience, as their primary focus would be understanding the functions of the MitraClip ^TM^ system. The use of an idealised model proved helpful in assisting participants in becoming acquainted with the device and the steps involved in the procedure, aligning with the initial phase of training. Nevertheless, feedback from participants indicated that the model’s anatomical precision was lacking, with misaligned structures and angles that did not accurately reflect the human heart, as observed in fluoroscopic imaging. As a result, the transfer of skills acquired during training to actual clinical practice could be more challenging, highlighting a pronounced learning curve.

The participants commented on the usefulness of the anatomical model when manoeuvring the device and knowing which borders and walls to avoid perforation or laceration of structures that could lead to cardiac tamponade in patients. This might contribute to the development of hand-to-eye coordination by combining movements on the system to simultaneously orient the CDS superiorly and laterally. A greater proportion of participants were less confident in positioning the trajectory of the CDS and recognising areas of interest on the MV to place the clip in the anatomical model compared with the idealised one. This could highlight areas in which training with this model can be improved further. For instance, the participant feedback in Table 4 suggested the use of visual feedback emulating 3D echo views and a pathological MV to increase the realism of the training.

According to these observations, training should be stratified according to operator expertise. Participants with no prior experience reported that the idealised model is very useful for learning and understanding manoeuvres. In contrast, the anatomical model is helpful for applying the fundamental steps of using the device to an enhanced platform for problem-solving and safely navigating within the confined borders of this model. Thus, rather than one model replacing the other, both could be integrated into different stages of training. However, to bring this to fruition, an examination of the candidate’s expertise is necessary to discern when to advance into the next stage of learning, so the training procedure needs to be standardised. Conventionally, training programs focus on the number of procedures or tasks completed by students, rather than evaluating the acquisition of skills. Thus, individualizing or stratifying training after discerning the baseline experience of students can increase confidence and promote efficient acquisition of skills [24, 25]. Additionally, an external examiner could assess and evaluate participant skills in each stage of the procedure. A checklist that evaluates participants’ movements, adherence to experimental guidelines and communication could be implemented in surgical simulation [26].

One limitation of this study is related to its small sample size, as well as not all participants were proceduralists, which limits the effectiveness of comparisons due to variability in grade or titles among participants of similar expertise. The study also faced methodological challenges. For instance, the availability of only one MitraClip™ kit meant that participants were influenced by their colleagues’ views while waiting to use the model, potentially biasing their evaluations and scores. A more rigorous approach, employing a randomised protocol and a larger participant group, would facilitate a more effective comparison. Future research should explore anatomical and pathological diversity, moving beyond a single patient cardiac model in the design phase to utilising statistical shape modelling for creating models that represent specific populations. Such an approach would enable the inclusion of pathological changes related to MR, such as enlargement of the left atrium and left ventricle.

To conclude, this study has shown that the anatomically realistic model is compatible with training with the MitraClip™ system and useful for increasing operator confidence, with participants largely agreeing that it is an effective simulator and should be integrated into clinical training. Nevertheless, this does not diminish the use of the idealised model, as participants with no prior experience found the model useful in familiarising themselves with the MitraClip™ device. Therefore, training should be stratified according to participant expertise, and the models should be distributed according to need. However, further research is necessary to evaluate the efficacy of the suggested improvements in increasing operator confidence and their impact on clinical outcomes.

### Electronic supplementary material

Below is the link to the electronic supplementary material.

Supplementary Material 1

## Data Availability

No datasets were generated or analysed during the current study.

## Abbreviations

CDS: Clip Delivery System
IAS: Inter-atrial septum
LA: Left atrium
LV: Left ventricle
MR: Mitral regurgitation
MV: Mitral valve
MVA: Mitral valve annulus
RA: Right atrium
SGC: Steerable Guide Catheter
m-TEER: Mitral Transcatheter Edge-to-Edge Repair
