# Supplementary material for: Effectiveness of a new 3D printed simulator for mitral transcatheter edge-to-edge repair in enhancing the confidence and procedural skills of the operator
Source: 3D Print Med. 2024 Aug 5;10:26. doi: 10.1186/s41205-024-00230-1 (PMC11299365; doi:10.1186/s41205-024-00230-1)
Supplement: Supplementary file 1 — Supplementary Material 1 [file 41205_2024_230_MOESM1_ESM.pdf]

APPENDIX

MitraClip Training Model Evaluation

What is your specialty?	
What is your current level of training?	

	Not confident				Highly Confident
Before this training, how confident are you with this procedure?	1	2	3	4	5

	Never before	A few times under supervision	Less than 1 year	1-5 years	More than 5 years
How long have you been carrying out this procedure for?	1	2	3	4	5

Post-Training

Please circle and rate for how accurate the following are compared to carrying out the procedure on a patient:

	Not Accurate				Highly Accurate
Carrying out the procedure on the model compared to in the catheterisation lab?	1	2	3	4	5
The height and angle of insertion above the mitral valve	1	2	3	4	5
The advancement of the catheter into the RA via the IVC	1	2	3	4	5

Please circle and rate for how confident you are for the following skills:

	Not confident				Highly confident
Transeptal crossing	1	2	3	4	5
Steering clip in LA	1	2	3	4	5
Positioning trajectory of clip	1	2	3	4	5
Recognising area of interest on Mitral Valve	1	2	3	4	5
Assessing position of clip above valve	1	2	3	4	5
Assessing orientation of clip above valve	1	2	3	4	5
Grasping leaflets	1	2	3	4	5
Closing a clip	1	2	3	4	5
Re-opening clip and repositioning	1	2	3	4	5
Removal of clip delivery system	1	2	3	4	5

How many minutes did it take for you to orientate within the LA?	
How many minutes did it take for you to complete the procedure (from insertion to clip deployment)?	

	Not effective				Highly effective
How effective is this model as a training simulator?	1	2	3	4	5

	Strongly disagree				Strongly agree
This model should be integrated into training for the procedure	1	2	3	4	5

	Not confident				Highly Confident
How confident are you with this procedure?	1	2	3	4	5

What did you find useful in this training model?

What could be improved in this training model?
